# Delivery of index-linked HIV testing for children: learnings from a qualitative process evaluation of the B-GAP study in Zimbabwe

**DOI:** 10.1186/s12879-023-08088-0

**Published:** 2023-04-26

**Authors:** Stefanie Dringus, Katherine Davis, Victoria Simms, Sarah Bernays, Nicol Redzo, Tsitsi Bandason, Rudo Chikodzore, Edwin Sibanda, Karen Webb, Getrude Ncube, Katharina Kranzer, Rashida A. Ferrand, Chido Dziva Chikwari

**Affiliations:** 1 Public Health, Environments and Society Department, London School of Hygiene and Tropical Medicine, Keppel Street, London, UK; 2 MRC Centre for Global Infectious Disease Analysis and the Abdul Latif Jameel Institute for Disease and Emergency Analytics, School of Public Health, Imperial College London, London, UK; 3 MRC International Statistics and Epidemiology Group, London School of Hygiene and Tropical Medicine, London, UK; 4 Clinical Research Department, London School of Hygiene and Tropical Medicine, London, UK; 5 Biomedical Research and Training Institute, Harare, Zimbabwe; 6 Global Health Department, London School of Hygiene and Tropical Medicine, London, UK; 7 School of Public Health, University of Sydney, Sydney, Australia; 8 Ministry of Health and Child Care Zimbabwe, Harare, Zimbabwe; 9 City Health Department, Bulawayo City Council, Bulawayo, Zimbabwe; 10 Organization for Public Health Interventions and Development, Harare, Zimbabwe; 11 Division of Infectious and Tropical Medicine, Medical Centre of the University of Munich, Munich, Germany

**Keywords:** HIV, Index-linked testing, Children, Implementation, Process evaluation, Barriers, Facilitators

## Abstract

**Background:**

Index-linked HIV testing for children, whereby HIV testing is offered to children of individuals living with HIV, has the potential to identify children living with undiagnosed HIV. The “Bridging the Gap in HIV Testing and Care for Children in Zimbabwe” (B-GAP) study implemented and evaluated the provision of index-linked HIV testing for children aged 2–18 years in Zimbabwe. We conducted a process evaluation to understand the considerations for programmatic delivery and scale-up of this strategy.

**Methods:**

We used implementation documentation to explore experiences of the field teams and project manager who delivered the index-linked testing program, and to describe barriers and facilitators to index-linked testing from their perspectives. Qualitative data were drawn from weekly logs maintained by the field teams, monthly project meeting minutes, the project coordinator’s incident reports and WhatsApp group chats between the study team and the coordinator. Data from each of the sources was analysed thematically and synthesised to inform the scale-up of this intervention.

**Results:**

Five main themes were identified related to the implementation of the intervention: (1) there was reduced clinic attendance of potentially eligible indexes due to community-based differentiated HIV care delivery and collection of HIV treatment by proxy individuals; (2) some indexes reported that they did not live in the same household as their children, reflecting the high levels of community mobility; (3) there were also thought to be some instances of ‘soft refusal’; (4) further, delivery of HIV testing was limited by difficulties faced by indexes in attending health facilities with their children for clinic-based testing, stigma around community-based testing, and the lack of familiarity of indexes with caregiver provided oral HIV testing; (5) and finally, test kit stockouts and inadequate staffing also constrained delivery of index-linked HIV testing.

**Conclusions:**

There was attrition along the index-linked HIV testing cascade of children. While challenges remain at all levels of implementation, programmatic adaptations of index-linked HIV testing approaches to suit patterns of clinic attendance and household structures may strengthen implementation of this strategy. Our findings highlight the need to tailor index-linked HIV testing to subpopulations and contexts to maximise its effectiveness.

## Contributions to the literature

- Index-linked HIV testing may lead to higher uptake and yield than blanket testing approaches and has been recommended by the World Health Organization.
- However, previous research into the process of implementing index-linked testing of children is limited. This recognised gap in the literature is a challenge to expansion of index-linked testing.
- Our findings highlight barriers to implementation along the index-linked HIV testing cascade of children, from reduced initial index clinic attendance to stockouts of test kits for diagnosing children. Knowledge of these barriers will allow optimisation of index-linked testing to support successful programme expansion.

## Background

Despite the global scale-up of paediatric antiretroviral therapy (ART) [1], children continue to experience disproportionate HIV-associated morbidity and mortality compared to adults [2]. Although children under 15 years were only 5% of the global population living with HIV in 2017, they represented 12% of HIV-related deaths [3]. This is due, in part, to late diagnosis of HIV and therefore delayed initiation of treatment among children [4]. Among those who survive infancy untreated, later ART initiation is associated with suboptimal immune reconstitution and risk of growth failure and organ damage [5]. Although prevention of mother to child transmission (PMTCT) programmes have been highly successful in preventing vertical transmission, many HIV-exposed infants are still missed and survive into childhood with undiagnosed HIV, and existing HIV testing strategies are insufficient in addressing the burden of undiagnosed HIV in children [6].

Given the relatively low prevalence of HIV among children, targeted strategies such as index-linked HIV testing have been recommended by the World Health Organization (WHO) to improve efficiency and reduce costs of HIV testing [7]. Index-linked HIV testing, whereby HIV testing is offered to children or sexual contacts of individuals living with HIV, has the potential to have higher uptake and yield and be more cost-effective than blanket testing approaches [8–10]. When this approach was implemented for children in Malawi, Kenya, Lesotho and Cameroon, it was found to have a higher yield of HIV compared to other testing strategies, although uptake of testing for children at risk of being HIV-positive varied substantially, ranging from 14 to 71% [10–12].

Studies have demonstrated highly variable rates of attrition across the entire index-linked testing cascade [10, 12, 13]. This cascade captures the flow of individuals through index-linked testing and is often conceptualised with steps including screening for eligible people living with HIV, provision of an offer of testing for children, acceptance of testing, and delivery of testing [11, 12, 14]. The diverse attrition rates noted in previous assessments of the cascade underscore the importance of understanding the context in which public health interventions are delivered.

The “Bridging the Gap in HIV Testing and Care for Children in Zimbabwe” (B-GAP) study evaluated uptake and yield of index-linked HIV testing for children aged 2–18 years in rural and urban communities in Zimbabwe [15]. While there was high uptake of the offer for index-linked HIV testing for children and adolescents (87.9%) and the strategy was acceptable to clients, only 60% of eligible children and adolescents eventually underwent testing [16, 17]. As part of the B-GAP study, a detailed process evaluation guided by the MRC framework [18], was conducted alongside the delivery of the intervention, exploring the implementation and receipt of the intervention. One part of the process evaluation is reported here, where we used implementation documentation to explore experiences of the field teams and project manager in implementing an index-linked testing program for children in Zimbabwe, and to describe barriers and facilitators to index-linked testing from their perspectives. Our results can inform programmes seeking to implement and scale-up index-linked testing about the key issues and strategies that they should consider. Further details of the process evaluation design [15] and results of other process evaluation components [17] are published elsewhere.

## Methods

### Index-linked HIV testing

The B-GAP study (protocol published, [15]) evaluated delivery of index-linked HIV testing for children in nine primary health clinics in Zimbabwe between January and December 2018. Six of the clinics were in Bulawayo (an urban setting) and three were in Mangwe district in Matebeleland South Province (a rural setting). Six healthcare workers (HCWs) trained on HIV testing delivered the intervention in the clinics. The HCWs worked in three teams of two at the first three clinics over a period of four months before moving to the next three clinics. This was done to align with three-monthly drug refills provided by the clinics to their clients and allowed an additional one month of follow up at each clinic.

Individuals with HIV attending the study clinics (indexes) were screened by the HCWs for eligibility. Indexes were eligible for inclusion in the study if they lived in a household with one or more children aged 2–18 years of unknown HIV status or with a known HIV negative result more than 6 months ago. Three options were offered to indexes who consented to have these children tested: (1) clinic-based testing; (2) community-based testing at home; or (3) an oral HIV self-test given to the index to test the child(ren) at home (known as caregiver provided HIV testing). Community-based HIV testing was delivered by a partner organisation which was already providing testing in the community, to avoid duplication of effort and parallel initiatives. All children eligible for HIV testing were followed up for up to 21 days to ascertain whether testing occurred, the test results where testing did occur, and in those where testing did not occur, the reasons for this.

### B-GAP process evaluation

#### Data sources

As part of the B-GAP study, a detailed process evaluation guided by the MRC framework [18] was conducted alongside the delivery of the intervention. One aspect of that process evaluation included using implementation documentation to qualitatively explore the experiences of the field teams and project manager who delivered the index-linked testing program, and to describe barriers and facilitators to implementation from their perspectives. To do this, data was drawn from four sources:

1. ***Weekly logs*** completed by the HCWs, which recorded data on clinic flow, recruitment, HIV testing, and stock levels using a semi-structured questionnaire on electronic tablets that were programmed using Open Data Kit. Only logs from the first six clinics were included in the analysis due to thematic saturation established in data from the initial clinics.
2. ***Minutes from monthly meetings*** held between the HCWs who delivered the intervention and the project co-ordinator to discuss study progress and troubleshoot challenges.
3. ***Incident reports*** completed by the project coordinator in the event of an incident that affected study progress or any adverse events, including corrective and preventive actions.
4. ***WhatsApp group chats*** between the HCWs and the project coordinator to communicate daily field activities.

#### Data analysis

**Fig. 1:**
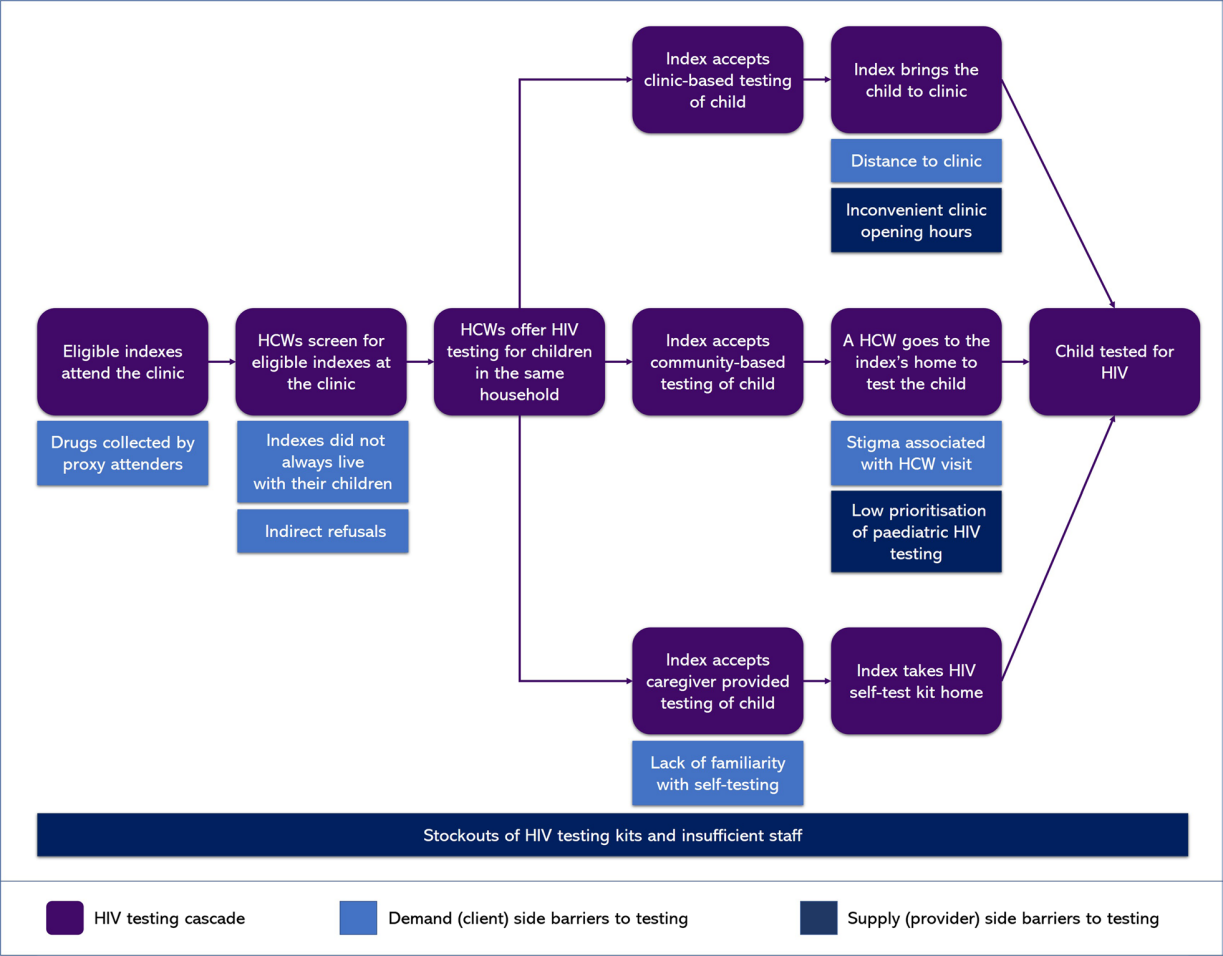
The HIV testing cascade and barriers to testing within the B-GAP study. Children could be tested through clinic-based testing, community-based testing or caregiver provided testing. HCW: healthcare worker

Data from the four data sources was extracted into Excel and read for familiarisation and open coding. A data-driven coding framework was developed, refined, and applied to each data source. Following initial coding, brief analytical memos were written to identify emerging key themes across each data source. These were then discussed by the first two authors SD and KD who developed a thematic framework by identifying over-arching analytical categories. The first two authors discussed these themes to enhance reflexivity [19]. After the themes had been refined, they were organised around an index-linked testing cascade framework. The framework was adapted from the initial B-GAP participant flow, using insights from other cascade frameworks [10–12, 14, 15]. It was conceptualised with nine steps, from clinic attendance to a child being tested for HIV (Fig. 1). Using the framework allowed exploration across the data set of how index-linked testing had been implemented and identification of key barriers and facilitators of implementation of index-linked HIV testing for children.

## Results

In total, 34 unique records from the four data sources were analysed to assess implementation. This included weekly logs from six clinics spanning February 2018 to July 2018 (n = 6); meeting minutes from four meetings between February 2018 and June 2018 (n = 4); incident reports between February 2018 and May 2018 (n = 22); and two WhatsApp chats. The first WhatsApp chat included all HCWs implementing the intervention, and the other was specifically for the rural district, as this area encountered distinct challenges.

The content of weekly logs varied from a sparse record of the number of clients seen to more detailed notes. Some HCWs recorded shorter logs than others, and the shortest logs coincided with particularly busy times in the clinics. The field teams discussed issues related to implementation and contextual factors in more detail during their monthly meetings. The WhatsApp chats provided a more informal record, including more detailed opinions on successes and challenges in implementation.

Five themes emerged from the analysis of the four data sources across the index-linked testing cascade (Fig. 1):

1. ***Eligible indexes attend the clinic—reduced clinic attendance of potentially eligible indexes***

For index-linked HIV testing to be effective, the approach requires indexes (individuals living with HIV) to attend clinics. In this study, individuals living with HIV often had their medication collected by others. This may have been done formally through participation in community ART refill groups (CARGs), where ART medication was picked up three-monthly by the CARG facilitator for each of its members, or by a family member or friend who collected ART on behalf of an index. ART collection by people other than indexes was observed in clinics in both urban and rural settings and was especially the case when clients resided in neighbouring countries for work, as was observed in Mangwe district, which is close to the Botswanan border:

*A lot of participants have their drugs collected by other people or the drugs are collected by one person on behalf of a treatment group. This means that the RAs are unable to screen these people. (Weekly log 02/2018)*

This reduced the number of potential indexes attending clinics, which resulted in missed opportunities to offer index-linked HIV testing for children.

2. ***HCWs screen for eligible indexes—indexes not living with their children***

The index-linked testing strategy used in B-GAP relied upon identifying eligible indexes, who reported living in a household with one or more children with an unknown HIV status or a known HIV-negative result more than six months ago. However, many indexes, particularly those attending urban clinics, reported that they did not live with their children. For example, this was encountered in a clinic that served predominantly migrant farm workers who had moved for work and therefore lived away from their families and children:

*Some indexes are farm workers and do not stay with their families resulting in a large number of indexes with no children. (Meeting minutes 03/2018)*

In more extreme cases, children of indexes lived in a neighbouring country. For example, one WhatsApp chat discussed how some indexes had children residing in Botswana, and thus unable to be offered testing.

3. ***HCWs screen for eligible indexes—soft refusals***

HCWs screening for eligible indexes also reported that they suspected that some individuals who did not want to take up index-linked HIV testing falsely claimed that no children lived in their household, i.e., a ‘soft refusal’. Occasionally, HCWs would later see one of these individuals in the clinic or in the community accompanied by children, supporting their suspicion that they were possibly avoiding being offered HIV testing for these children. Although this was not often reported in the log reports, it was discussed in detail during team meetings.

4. ***Delivery of testing—difficulties attending clinic-based testing, low prioritisation and stigma of community-based testing, little familiarity with caregiver provided testing***

When indexes accepted testing, there were challenges encountered with bringing the child(ren) in contact with HIV testing, regardless of the HIV testing method chosen.

**Clinic-based testing**

Of the indexes that opted for clinic-based testing of children, clinic appointments were frequently missed in both urban and rural settings. This was mainly due to transport difficulties and distance from the clinic. As expected, missed appointments were reported more often in rural settings:

*I have an additional household that said it’s so difficult for her to bring the kids because there is no transport to the place yet it’s so far. (WhatsApp chat—06/2018)*

In addition, relocation was common, and this served as an additional challenge to increasing the coverage of HIV testing:

*Indexes who are tenants and always moving and relocating, they may have moved away from the clinic catchment area but still getting their medication at the clinic. (Meeting minutes 03/2018)*

Other reasons for missing clinic appointments for testing included a lack of time or convenient clinic operating hours in both rural and urban settings. This was particularly the case for children who were enrolled in school or helping at home:

*Some of the households are bringing children for testing past the 21 days stating that children go to school midweek, and they need them to assist finishing with harvesting during weekends. (Meeting minutes 06/2018)*

**Community-based testing**

The process evaluation found that partner organisations tasked with delivering community-based HIV testing in homes had insufficient resources and other competing priorities, and therefore index-linked HIV testing for children was not consistently delivered, particularly in the rural settings:

*Community testing is still a challenge as [partner organisation] keeps promising that someone will come but ever since we started in [Clinic 1], no one has come for community testing. (Meeting minutes 06/2018)*

*They [partner organisation] said they are short staffed, and they will not be able to do household visits. They did not test children. (WhatsApp chat 05/2018)*

In some instances, partner organisation aims were perceived to be different from the aims of B-GAP. For example, targets to maximise HIV self-testing, and a lack of resources resulted in a lesser priority of community-based testing of children:

*There is a lack of prioritization of B-GAP from [partner organisations’] end as some locator forms have come back without convincing evidence of clients follow up (Meeting minutes 04/2018)*

To try to remedy this, a dedicated B-GAP project HCW was assigned to conduct community-based HIV testing in homes in the rural area initially, and then later also in the urban study areas. Consequently, children who did not attend their facility testing appointments could be followed up actively in the community. Subsequently, HIV testing uptake among children whose index had initially opted for clinic-based testing increased, as noted by one of the HCWs:

*Community testing has helped in closing a lot of files. We have been going to the community and testing those who had opted for clinic testing but did not come (Weekly log 05/2018)*

Community-based HIV testing, even when fully operational, was not without challenges. Despite the approach being valued for its convenience, concerns about stigma persisted. HCWs recorded in their weekly logs and discussed at the supervisory meetings how indexes feared that the appearance of B-GAP staff at their home could potentially lead to negative reactions and gossip from friends, family, and neighbours:

*[community-based testing] is generally not widely selected as some find it weird to get a health worker come and visit them (Weekly log 03/2018)*

*Community testing remains low in Mangwe because of the perceived stigma of neighbours seeing a [partner organisation] car arriving at one’s homestead (Meeting minutes 03/2018)*

Having to balance convenience with the possibility of stigma meant that community-based HIV testing was found not to have been preferred initially but became a more appealing option when the clinic was not practically accessible.

**Caregiver provided testing**

As a third option, indexes who were parents or legal guardians of a child could opt to take HIV oral tests home and test their child(ren). HCWs reported that indexes rarely chose this option. Those who did choose it had usually struggled to access the clinic, in a time when community-based testing was not operational:

*Very few select it. For those few that select it, they have school children who come from school after 4 or 5[pm]. For the other few, the places are hard to reach as public transport leave them far. (WhatsApp chat 05/2018)*

The other, rare, instances where indexes chose caregiver provided testing was when they had prior experience with the method, as explained during one of the group meetings:

*The general feel about self—testing so far is that people are not used to it yet and the few people that have selected self-testing at some of the sites are people who have been exposed to it previously (Meeting minutes 04/2018)*

HCWs felt that caregiver provided testing was less appealing to indexes because it required them to read instructions.

*Caregiver provided testing is the least preferred of the testing options. This could be attributed to literacy levels of the clinic's catchment area (Weekly log 06/2018)*

5. ***Across the cascade—stock and staffing challenges***

Delivering index-linked testing requires stock of HIV testing kits and supplies and suitable staffing levels at the clinics. However, logistical challenges associated with this were frequently encountered and affected delivery. Some of these problems were related to the long-standing economic crises in Zimbabwe. For example, in August 2018, clinic staff went on strike resulting in several days of clinic closures, and impacting on testing:

*The week started slowly as the clinic staff was on a strike, this did not affect index screening as they were attending emergencies and OI patients only. The testing side was affected however [our HCWs] had to step in and do the testing. (Weekly log 04/2018)*

HCWs from the BGAP study supported routine clinical care and provided HIV testing when clinics were short-staffed due to strikes, or non-clinical administrative workloads such as stocktaking. Additionally, the national pharmaceutical supplier had difficulties managing their supply chain, and as a result, stock out of test kits occurred in some clinics, mainly in the rural district:

*[national pharmaceutical supplier] has the test kits but we do not understand where the backlog is with getting the test kits delivered to the site. This issue has not yet been resolved although the clinic is now using test kits that the project bought (a box of 20 test kits) delivered on March 22nd (Weekly log 04/2018)*

*This side is now becoming hectic. Today the nurses were packing their stock from [national pharmaceutical supplier] and there was no one doing HIV testing. Yet many people answered to the follow up calls I made on Friday. Therefore, we missed all indexes that came today but advised them to just bring all the kids not tested or tested more than six months ago (WhatsApp chat 06/2018)*

## Discussion

This manuscript reports the operational challenges at various stages of the index-linked testing cascade, which need to be considered for effective implementation and scale up. Five main themes were identified. Firstly, there was lower clinic attendance among potentially eligible indexes due to collection of HIV treatment by proxy individuals. Secondly, some indexes reported that their children did not live in their household, as result of high levels of mobility. In addition, there were thought to be cases of ‘soft refusal’, when potential indexes falsely claimed not to have children in their household. Furthermore, delivery of HIV testing was constrained by difficulties attending health facilities for clinic-based testing, stigma around community-based testing, and low familiarity with caregiver-provided oral HIV testing. Lastly, stockouts and inadequate staffing also limited delivery of index-linked HIV testing.

The first theme that was identified was the reduced clinic attendance of potentially eligible indexes. This resulted in fewer opportunities for indexes to be offered HIV testing for their children, and ultimately possibly fewer children being tested. In recent years, there has been a move towards differentiated service delivery to make HIV care more client-centred and reflect the preferences and expectations of people living with HIV, while reducing the burden on the health system [20]. CARGs and proxy collection of ART refills are convenient for clients and reduce the pressure on health facilities but reduce contact with healthcare providers where broader aspects of HIV care, including testing of HIV-exposed children, can be discussed. This can be mitigated by ensuring that testing of children is actively addressed when a client does attend for a clinic visit by continuous follow up through CARGs and when indexes attend the facility for clinical reviews (once every 6 months in Zimbabwe). Alternatively, the feasibility of index-linked testing being extended beyond clinics and being discussed and potentially offered to individuals within CARGs, needs to be explored. While in-country guidance recommends testing of all biological children of ART clients, implementation fidelity of this in large scale programs with many competing demands is yet to be evaluated [21]. More broadly, while community-based models of delivery of ART and adherence support are increasingly being implemented, programmes are increasingly cognisant of broader aspects of HIV management including, but not restricted to, testing of children and sexual partners. One example is screening for comorbidities, such as cervical cancer. These aspects have now been incorporated into an annual check-up visit that integrates HIV testing for contacts, HIV viral load monitoring and a clinical check-up with ART delivery in community-based settings [21]. Our findings underscore the importance of implementation fidelity monitoring in context, within and between populations for evidence-based and responsive programs.

At the next step in the index-linked testing cascade, when indexes accessed the clinic, we found that some indexes were not living with their children, as in the case of migrants. Healthcare delivery is particularly challenging among mobile and migrant populations and special counselling and active follow-up may be required to ensure that indexes whose children may not be with them do get their children tested. In the Zimbabwean context, children also spend extended time with relatives who are not their parents or primary caregivers and may attend boarding school [22]. Children changing caregivers has been reported to be a barrier to successful implementation of index-linked testing for children in other studies [23, 24].

When index-linked HIV testing *was* offered to indexes whose children were living with them, ‘soft’ or indirect refusals through denial that they had children eligible for testing was reported. Previous studies have highlighted some reasons why HIV testing for children may not be taken up including deductive or indirect disclosure of parental HIV status, or the child being stigmatised if his or her status is revealed [10, 17, 23, 25]. HIV testing of children needs to be accompanied by ongoing support for the disclosure process both for the caregiver and for the child. In the B-GAP study, HIV testing was offered to all children in the household of the index, and indexes may have either been uneasy or unable to consent (due to legal reasons) for a child to be tested whom is not biologically theirs [12, 17].

Further in the cascade, delivery of HIV testing was associated with a range of challenges. Consistent with findings from other studies [10, 26–29], despite initial high uptake of index-linked HIV testing, indexes faced difficulties attending health facilities with their children. Appointments for clinic-based HIV testing were missed due to distance from the clinic and difficulties accessing transport, a lack of time or inconvenient clinic opening hours. To mitigate against these challenges, the B-GAP study provided an alternative option for community-based HIV testing in the home by HCWs from partner organisations [15]. However, community-based testing of children by partner organisations was not reliable, in part due to low prioritisation of index-linked testing and limited existing program resources. In response, the study protocol was modified to employ dedicated HCWs for community-based HIV testing. Despite these efforts and strengthened identification of children in need of HIV testing, 40% of eligible children were not tested in the study, indicating remaining gaps in reaching children and adolescents not addressed by the study. Providing support to indexes to access clinics for testing of children, such as covering transportation costs and having flexible opening times for testing of children, should be a consideration for HIV testing programs targeted towards children. This can be supported by, and work in partnership with, ongoing community-based testing and follow up.

It is important to recognise the role of stigma in constraining delivery of HIV testing. Notably, in the B-GAP study, the most popular choice for testing was clinic-based testing rather than community-based testing [16]. This contrasts with findings from a study which provided index-linked testing for children and young people (aged 1–24 years) in Malawi where 88% of indexes opted for community-based testing [12]. This may reflect fear of stigma that might have occurred if households were visited for HIV testing, especially if they were called on by a community HCW in a vehicle branded with their organization’s name, which may disclose the reason for the visit and stigmatise the household. Where possible, community testing interventions should avoid the use of branded vehicles and clothing for staff to mitigate against potential stigma. Interestingly, many of those who had initially selected clinic-based testing did, however, have their children tested at home later, when they were followed up by B-GAP staff after having missed facility appointments. This suggests that community-based testing at home by HCWs may serve as a “mop-up” HIV testing strategy and address some of the challenges that individuals may face in accessing clinic-based testing for their children. Indexes may also have felt more comfortable being visited by B-GAP HCWs whom they had already met when they were initially offered testing in the facility. Importantly, offering indexes a choice of testing location may improve uptake and acceptability [30].

Community-based HIV testing does require additional resources and there may be concerns regarding cost-effectiveness especially when it is implemented for children who have a relatively low HIV prevalence compared to adults [31]. One possible approach is the use of HIV self-testing with enhanced support to enable caregivers to test their children. Within B-GAP, it was found that caregivers were able to test their children correctly [32], but the uptake of this method was low, which likely reflects lack of familiarity with caregiver provided oral testing and possibly a desire for support [16, 32]. It is however an approach that could be used to address some of the barriers in accessing clinic-based testing, as well as concerns of stigma associated with visits by a HCW in the community. This would require sensitisation of communities to increase awareness and engender confidence among caregivers in testing their own children.

Finally, across the testing cascade, stock and staffing challenges were identified as a problem. For example, stockouts of testing kits impeded effective implementation of index-linked HIV testing for children. Other studies have reported similar findings, including, notably, for other paediatric HIV testing approaches such as provider-initiated HIV testing and counselling as well as early infant diagnosis within PMTCT programmes [25, 33–35]. Indeed, studies have shown that in the event of periodic shortages, HIV testing kits have been prioritised for adults [26]. Staffing constraints were a further barrier to delivery of index-linked HIV testing. We had originally anticipated evaluating index-linked HIV testing delivered by routine clinic staff. It became clear, however, that this would not be feasible, and instead the project recruited HCWs who were responsible for delivery of index-linked HIV testing and supported clinic staff. These dedicated B-GAP study HCWs also took up other clinic duties when there was industrial action by clinic staff. Adding interventions to health systems without adequately considering human resources and strengthening supply chains is retrogressive for programme success. A further important consideration for scale-up of this approach is adequate training and support for HCWs; when HCWs lack the skills to counsel caregivers and their children about HIV testing, they do not offer it [26, 36].

A strength of this study was the use of data from multiple sources collected throughout the delivery of the intervention. These were triangulated to understand implementation [18]. The tools were created to both prompt HCWs to reflect on the delivery of the intervention and to report challenges, which would prompt real-time problem-solving and improvements in intervention delivery. However, this may have resulted in an emphasis on barriers and challenges rather than facilitating factors and positive experiences [18]. While the B-GAP study HCWs’ position gave them an excellent vantage point to understand operational issues, a limitation of this evaluation is that they rarely assessed or reflected on their own behaviour, and how that shaped implementation. The perspectives of indexes have been reported elsewhere [17], but perspectives of other stakeholders including routine clinic staff may contribute to understanding how index-linked HIV testing can be effectively integrated into HIV care delivery.

## Conclusions

Available evidence suggests that index-linked HIV testing is an efficient intervention and could be leveraged to close the gap in paediatric HIV testing. Index-linked HIV testing for children is increasingly being integrated into wider HIV policies for children, but it is critical to find ways to limit attrition at each point along the HIV testing cascade to achieve optimum outcomes. Reduced attendance of indexes at clinics, indexes not living with their children and soft refusals may be important sources of early attrition. Difficulties in accessing clinics, stigma around community-based testing and a lack of familiarity with caregiver-provided oral HIV tests can be key challenges later in the process. In addition, poor stock and staffing levels could trigger attrition across the cascade and should be addressed to improve testing for children. In addressing challenges, it is also important to remember that one size does not fit all. Providing different options for HIV testing that consider diverse local contexts, addressing supply chains and human resources and strengthening partnerships between facility and community-based initiatives will be key to scaling up delivery of index-linked HIV testing and more broadly reducing the burden of undiagnosed HIV in children. Finally, index-linked testing for children should be accompanied by robust approaches for linkage to care and treatment.

## Data Availability

The anonymised datasets apart from WhatsApp chats used during the study are available from the corresponding author on reasonable request.

## Abbreviations

ART: Anti-retroviral therapy
B-GAP: Bridging the Gap in HIV Testing and Care for Children in Zimbabwe
CARG: Community ART refill group
HCW: Healthcare worker
PMTCT: Prevention of mother to child transmission
WHO: World Health Organization
